# Transient plasma viral rebound after SARS-CoV-2 vaccination in an exceptional HIV-1 elite controller woman

**DOI:** 10.1186/s12985-023-02086-z

**Published:** 2023-06-13

**Authors:** L. Di Girolamo, M. Ferrara, G. Trevisan, B. M. Longo, T. Allice, E. Burdino, F. Alladio, S. Fantino, G. Di Perri, A. Calcagno, S. Bonora

**Affiliations:** 1 Unit of Infectious Diseases, Department of Medical Sciences, University of Torino, Corso Svizzera 164, Turin, 10149 Italy; 2 Laboratory of Microbiology and Virology, Amedeo di Savoia Hospital ASL Città di Torino, Turin, Italy

**Keywords:** HIV, Elite controller, SARS-CoV-2 mRNA vaccines, HIV-RNA rebound, Total HIV-DNA

## Abstract

**Background:**

Elite controllers are able to control viral replication without antiretroviral therapy. Exceptional elite controllers do not show disease progression for more than 25 years. Different mechanisms have been proposed and several elements of both innate and adaptive immunity are implicated. Vaccines are immune stimulating agents that can promote HIV-RNA transcription; transient plasma HIV-RNA detectability has been described within 7–14 days after different vaccinations. The most reliable mechanism involved in virosuppressed people living with HIV is a generalized inflammatory response that activates bystander cells harboring latent HIV. So far no data about viral load increase in elite controllers after SARS-CoV-2 vaccination are reported in literature.

**Case presentation:**

We report the case of a 65-year-old woman of European ancestry, diagnosed with HIV-1/HCV co-infection more than 25 years ago. Since then, HIV-RNA remained undetectable and she never received ARV therapy. In 2021 she was vaccinated with mRNA-BNT162b2 vaccine (Pfizer-BioNTech®). She was administered with three doses in June, July and October 2021, respectively. The last available viral load was undetectable in March 2021. We observed an increase of VL at 32 cp/ml and 124 cp/mL, two and seven months after the second vaccine dose, respectively. During monthly follow-up, HIV-RNA gradually and spontaneously dropped becoming undetectable without ARV intervention. COVID-19 serology was positive with IgG 535 BAU/mL, showing response to vaccination. We measured total HIV-DNA at different time-points and we found it detectable both at the time of the higher plasma HIV-RNA (30 cp/10^6 PBMCs) and when it was undetectable (13 cp/10^6 PBMCs), in reduction.

**Conclusions:**

This case is the first report, to our knowledge, describing a rebound of plasma HIV-RNA in an elite controller after three doses of mRNA-BNT162b2 vaccine for SARS-CoV-2. Concomitantly with a spontaneous reduction of plasma HIV-RNA ten months after the third dose of mRNA-BNT162b2 vaccine (Pfizer-BioNTech®) without antiretroviral therapy intervention, we observed a reduction of total HIV-DNA in peripheral mononuclear cells. The potential role of vaccinations in altering HIV reservoir, even in elite controllers when plasma HIV-RNA is undetectable, could be a valuable aspect to take into account for the future HIV eradication interventions.

## Background

Elite controllers (EC) are a rare group of HIV-infected individuals with the ability to spontaneously control HIV replication in the absence of antiretroviral therapy [1, 2]. Controllers are identified and classified either as viremic or elite, according to their plasma viral load (VL); the first ones have sustained measurement of 50-2000 RNA copies/ml after six months of infection, whereas the second ones have undetectable VL [3]. Exceptional elite controllers are subjects who maintain EC characteristics without disease progression for more than 25 years [4].

Different mechanisms have been proposed and several elements of both innate and adaptive immunity are implicated even though the specific mechanisms by which elite controllers achieve control remain undefined [2].

These include defective HIV-1 variants, different HLA gene expression with consequent different CTL and NK response to HIV-envelope proteins epitopes, different sequences of HIV co-receptor and their ligands, such as 𝝙32 deletion and RANTES expression, the presence of poly-functional CTL response pointing to the higher relevance of its quality compared to its quantity. Conflicting studies exist about neutralizing antibodies in HIV disease progression. Moreover, different studies suggest that proviral amplification is significantly lower in EC and that the integration of intact proviral sequences in repressive chromatin locations in chromosomal sites are suggestive of deep latency. These features define the “block and lock” mechanism of viral control that silences proviral gene expression [2, 3, 5, 6].

Due to their immune-stimulating nature, vaccines have been evaluated as possible agents for “shock and kill” strategies to activate HIV transcription and virion production in order to eliminate the reservoir [7, 8]. The most accredited mechanism involved in increased HIV transcription, observed after vaccine administration in virosuppressed people living with HIV (PLWH), is a generalized inflammatory response with cytokine production that activate bystander cells harboring latent HIV, or activation of infected vaccine-specific T-cells. Transient plasma HIV-RNA detectability has been described within 7–14 days after vaccination for influenza, pneumococcus, tetanus, hepatitis B and cholera in antiretroviral therapy (ART)-suppressed PLWH [9, 10]. On the other hand, in another report of participants on suppressive ART, a significant increase of median cell associated HIV-RNA levels, an overall T-cell activation and HIV-specific CTL function was observed, but no change in total HIV-DNA and plasma HIV-RNA was found [11]. A recent case report described a transient increase in VL in an ART-suppressed 65-year of age patient 28 days after the first dose of mRNA-1273 vaccine (Moderna Biotech). Plasma HIV-RNA was found to be undetectable 14 days after the VL increase [11, 12]. To date, no data about VL increase in HIV elite controllers after SARS-CoV-2 vaccination are reported in literature.

## Case presentation

Here we report a case of a woman (65 years of age) of European ancestry, diagnosed with HIV-1/HCV co-infection at the age of 37. Drug addiction from 1975 to 1990 and heterosexual intercourse were her main risk factors. Baseline immunological assessment CD4 + and CD8 + T-cell count were respectively 658 cells/µL and 679 cells/µL with a CD4+/CD8 + ratio of 1.0 and, according to the current international HIV guidelines at the time, antiretroviral therapy (ARV) was not carried out. No other co-infections or comorbidities were reported apart from anxiety and depressive disorders.

In March 1998 the first HIV-RNA Polymerase Chain Reaction (PCR) was performed, showing HIV-RNA < 200 copies/mL, genotype B, wild type. Since then, ARV therapy was not started due to the optimal CD4 + T-cell count and the concomitant viral suppression (< 20 cp/mL), spontaneously maintained during the following years.

In 2001 the patient was treated for HCV with interferon (IFN) plus ribavirin with a sustained virologic response.

In September 2017 she was vaccinated with pneumococcal and tetanus-diphtheria vaccine, and we observed a viral load increase at 93 cp/mL eight months after the infusion. In that occasion immunological status remained unaltered and viral load spontaneously resulted to be undetectable the following month.

Since then, she was regularly followed and refused to initiate any ARV therapy even though various reports in literature underlined the importance of beginning therapy in the elite controller population [13].

In 2021, due to severe acute respiratory syndrome coronavirus 2 (SARS-CoV-2) pandemic, she was vaccinated with mRNA-BNT162b2 vaccine (Pfizer-BioNTech®) in the context of the Italian vaccination campaign. The first dose was administered on June 24th 2021 and the second on July 25th 2021. The last available VL was undetectable on March 24th 2021. During the regular semestral follow-up, in September 2021, we observed an increase of plasma HIV-RNA at 32 cp/mL, precisely two months after the second dose of vaccine.

Afterwards, a third dose of mRNA-BNT162b2 vaccine was scheduled on October 2nd. Due to detectable VL, it was decided to monthly control the immuno-virological status at the Outpatient Clinic. The highest HIV-RNA viremia was found to be 124 cp/mL on January 13th 2022, seven months after the first vaccine dose. On that occasion we measured total HIV-DNA by means of HIV-1 DNA Test PRO which allows the detection and quantification of total HIV-1 DNA, M group in whole blood samples and PBMC, resulting in 30 cp/10^6 PBMC. No data were available for reservoir measurement around the time of the first dose of vaccine. Total HIV-DNA was previously measured in 2017 with a home-made technique, and resulted to be undetectable.

During monthly follow-up controls, HIV-RNA gradually and spontaneously dropped, until it became undetectable on August 10th 2022. Concomitantly we observed a slight reduction of total HIV-DNA resulting in 13 cp/10^6 PBMC, below the limit of quantification of 20 cp/10^6 PBMC.

We performed COVID-19 serology, resulting in positive IgG 535 BAU/mL 10 months after the third dose of vaccine because the SARS-CoV-2 serology was not available immediately after the first/second dose. Throughout the follow-up, CD4 + and CD8 + T-cells and CD4+/CD8 + ratio, measured in September 2021, February 2022, March 2022 and November 2022, remained unaltered and the patient remained asymptomatic with a normal blood chemistry.

## Discussion

This case is the first report, to our knowledge, to describe a rebound of plasma HIV-RNA in an elite controller individual whose viremia was spontaneously controlled without ART intervention after three doses of mRNA-BNT162b2 vaccine for SARS-CoV-2. In the literature, there are reports of patients with stable ARV treatment who show transient increase in plasma HIV-RNA after SARS-CoV-2 vaccination [2, 11, 12, 14]. The timing from vaccination to HIV-RNA rebound is still debated, but most studies demonstrating the post vaccination plasma HIV-RNA increase, report 1–2 weeks from the vaccine injection [11, 12]. In our case, we do not have collected data until two months after the second dose. We ruled out the other potential causes of viral rebound such as other vaccinations, reinfection from unprotected sexual intercourse with HIV positive partners or other coinfections. Moreover, the patient did not report COVID-19 disease either before or during the time of the strict follow-up.

In ART suppressed PLWH, standard vaccinations are able to start a generalized inflammatory response resulting in a transient overall increase in memory CD4 + and CD8 + cells as well as activated CD4 + CD38 + and CD8 + CD38 + cells. On the other hand, in elite controllers, the mechanism of the inflammatory response following a vaccination is not known, unlike those with a persistent HIV load.

At the time of the achievement of plasma HIV-RNA undetectability, ten months after the third dose of mRNA-BNT162b2 vaccine (Pfizer-BioNTech®), we observed a concomitant slight reduction of total HIV-DNA in PBMCs.

This could suggest that the mechanisms of virological control in this patient are still working despite the inflammatory stimuli given by vaccination, in this case. On the other hand, it is already known that deep viral latency is not completely permanent or irreversible but, more likely, is a dynamic process and occasional bursts of viral transcription may occur despite genomic and epigenetic integration site features [6].

In conclusion, the interplay of mRNA vaccines, the immune system, and latent HIV infection in reservoir is yet to be thoroughly understood. Our findings add new perspectives in the clinical context of the PLWH vaccination. For instance, PLWH should be more strictly monitored close to the vaccination time, as virological rebounds could have serious implications (higher rate of HIV transmission and emergence of drug resistance). Moreover, the role of vaccinations in altering the latent HIV reservoir even in elite controller individuals could be a valuable option to take into account for future HIV eradication interventions.

## Data Availability

All data generated or analyzed during this study are included.

## List of abbreviations

ART: Antiretroviral therapy
EC: Elite controllers
IFN: Interferon
PCR: Polymerase Chain Reaction
PLWH: People living with HIV
SARS-CoV-2: Severe acute respiratory syndrome coronavirus 2
VL: Viral load
